# A rationally engineered yeast pyruvyltransferase Pvg1p introduces sialylation-like properties in neo-human-type complex oligosaccharide

**DOI:** 10.1038/srep26349

**Published:** 2016-05-19

**Authors:** Yujiro Higuchi, Sho Yoshinaga, Ken-ichi Yoritsune, Hiroaki Tateno, Jun Hirabayashi, Shin-ichi Nakakita, Miho Kanekiyo, Yoshimitsu Kakuta, Kaoru Takegawa

**Affiliations:** 1 Department of Bioscience and Biotechnology, Faculty of Agriculture, Kyushu University, 6-10-1 Hakozaki, Fukuoka 812-8581, Japan; 2 Biotechnology Research Institute for Drug Discovery, National Institute of Advanced Industrial Science and Technology, Central-2, 1-1-1, Umezono, Tsukuba, Ibaraki 305-8568, Japan; 3 Department of Functional Glycomics, Life Science Research Center, Kagawa University, Miki-cho, Kagawa 761-0793, Japan

## Abstract

Pyruvylation onto the terminus of oligosaccharide, widely seen from prokaryote to eukaryote, confers negative charges on the cell surface and seems to be functionally similar to sialylation, which is found at the end of human-type complex oligosaccharide. However, detailed molecular mechanisms underlying pyruvylation have not been clarified well. Here, we first determined the crystal structure of fission yeast pyruvyltransferase Pvg1p at a resolution of 2.46 Å. Subsequently, by combining molecular modeling with mutational analysis of active site residues, we obtained a Pvg1p mutant (Pvg1p^H168C^) that efficiently transferred pyruvyl moiety onto a human-type complex glycopeptide. The resultant pyruvylated human-type complex glycopeptide recognized similar lectins on lectin arrays as the α2,6-sialyl glycopeptides. This newly-generated pyruvylation of human-type complex oligosaccharides would provide a novel method for glyco-bioengineering.

*N*-linked protein glycosylation mediates a variety of cellular processes, such as protein interaction and cell-cell communication, dysfunction in which leads to diseases in mammals^1^,^2^,^3^,^4^,^5^. *N*-glycans are modified by a versatile group of glycosyltransferases, the end chains of which are attached to distinctly different molecules in various organisms. For instance, in mammalian cells, sialic acid gets attached to the terminal oligosaccharides^6^,^7^. In contrast, model yeasts *Saccharomyces cerevisiae* and *Schizosaccharomyces pombe* harbor phosphate and pyruvate, respectively, on their cell surface oligosaccharides^8^,^9^,^10^,^11^,^12^,^13^,^14^,^15^. Pyruvylation has been observed in both prokaryotic and eukaryotic organisms, such as *Bacillus anthracis*, marine sponge *Microciona prolifera* and red seaweeds *Cryptonemia seminervis* and *Laurencia filiformis*^16^,^17^,^18^,^19^,^20^. In *Escherichia coli*, pyruvylation of capsule structure and colonic acid, mediated by WcaK protein, is crucial for these cells to escape the attack of the immune system^21^. It has been reported that pyruvyltransferase PssM is required for the symbiosis of *Rhizobium leguminosarum bv. viciae*-*Pisum sativum*^22^. Most importantly, pyruvylation provides negative charges on the cell surface, which is crucial for intercellular interactions in *M. prolifera*^16^ and *S. pombe*^23^,^24^. Since attachment of sialic acid also provides negative charges on the cell surface, pyruvylation or sialylation on terminal oligosaccharides may confer similar functional effects^9^.

The molecular mechanism of pyruvylation has been studied well in the model yeast *S. pombe*. Accordingly, pyruvate is added to the oligosaccharides of glycoproteins by the pyruvyltransferase Pvg1p, which is localized to the Golgi membrane. In the Golgi, Pvg1p relies on two transporters, Pet1p and Pet2p, for the supply of its substrate phosphoenolpyruvate (PEP), which is transported from the cytoplasm into the Golgi lumen^25^. We previously demonstrated that Pvg1p could specifically transfer a pyruvate group onto the α-linked galactose (α-Gal) residues of oligosaccharides^26^.

In the present study, in order to elucidate the pyruvyl transfer mechanism of Pvg1p and also to understand the structural basis of its substrate specificity, we first determined the crystal structure of Pvg1p at a resolution of 2.46 Å. The folding pattern of Pvg1p resembled that of the type-B glycosyltransferase, including sialyltransferases. Based on our *in-silico* models of the substrate-enzyme complex and results of mutational analyses, we presented evidence for the underlying structural basis for the substrate specificity of Pvg1p. In addition, by rational protein engineering of Pvg1p, we were able to create a Pvg1p mutant that could transfer pyruvate moiety onto a human-type complex oligosaccharide efficiently. Importantly, we observed that the molecular properties of the pyruvylated human-type complex glycopeptide were similar to those of the α2,6-sialyl glycopeptide, suggesting that pyruvylation can mimic sialylation. Based on our results, we believe that this modification offers a strategy for generating novel glycopeptides.

## Results

### Crystallization of Pvg1p and analysis of crystal structure

For crystallization, we purified recombinant Pvg1p, which was expressed in *E. coli*, using a combination of Ni^2+^-affinity chromatography and gel filtration chromatography, and confirmed its purity by SDS-PAGE (Supplementary Fig. S1). The purified Pvg1p was crystallized in the presence of Zn^2+^. Data collection and refinement statistics are shown in Table 1. However, despite our several attempts to co-crystalize Pvg1p with its substrates PEP and β-Gal-*p*-nitrophenyl (*p*NP), we could not find any crystal of Pvg1p to which both substrates were bound.

**Figure 1:**
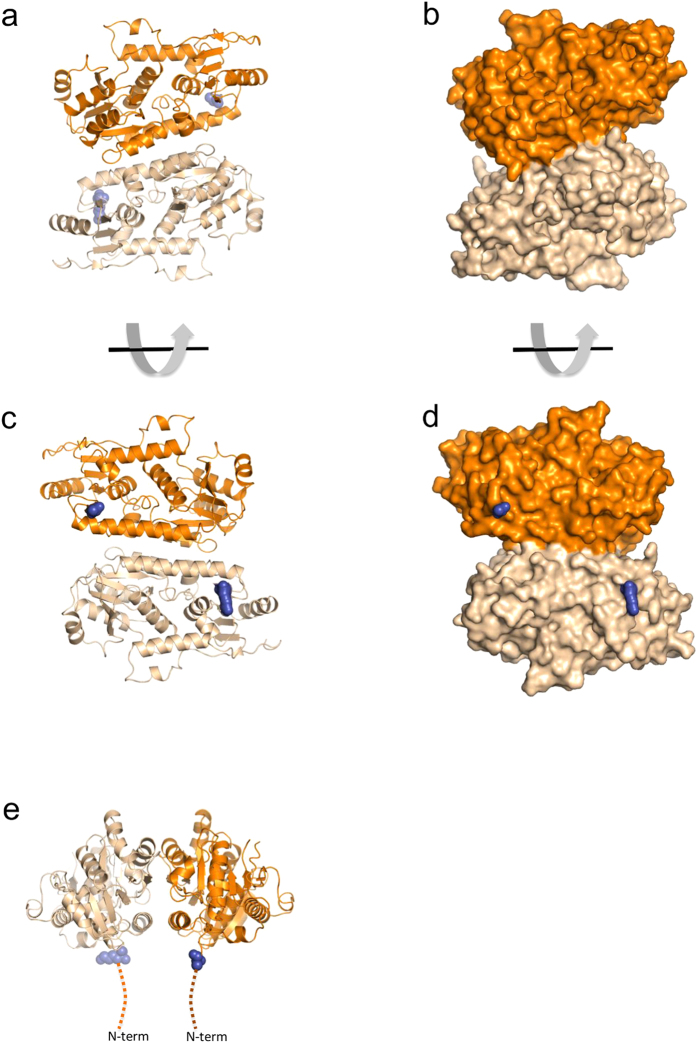
Dimeric complex of Pvg1p. Protomers A and B are coloured orange and weak orange, respectively. (**a**–**d**) Views of the Pvg1p dimer looking down the homodimer two-fold axis. (**e**) The side view of the dimer. Hypothetical positions of the N-termini of both protomers are sketched as dark blue in the figures. The Golgi membrane is located ipsilateral to the N-termini.

**Figure 2:**
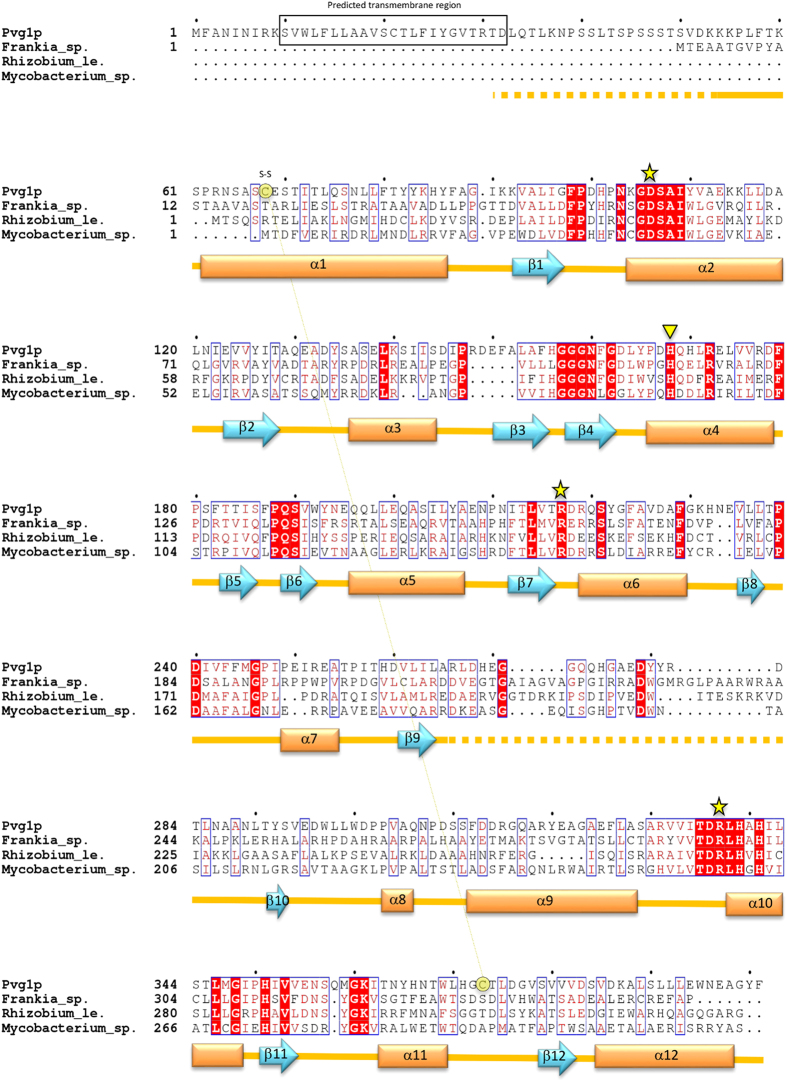
Sequence alignment of Pvg1p and its homologs. Alignment of amino acid sequences of Pvg1p and its homologs from *Frankia* sp. (accession number, ABW10208.1), *Rhizobium leguminosarum* (accession no. CAK09136.1) and *Mycobacterium* sp. (accession no. ABG09219.1). D32-F401 portion of Pvg1p was used for crystallization. The annotated secondary structures of Pvg1p are indicated below the aligned sequences (arrows: β-strands, boxes: α-helices). Disordered regions are indicated by dashed-lines. The disulfide bond (C68-C373), revealed from the crystal structure analysis of Pvg1p, is also shown. Proposed catalytic residues (D106, R217 and R337) are marked with yellow stars. Yellow inverted triangle shows the H168 residue that was subjected to mutation analysis. Predicted transmembrane region is boxed in black. All residues important for the pyruvyltransferase activity of Pvg1p are conserved among the homologs.

Diffraction analysis of the crystal revealed a structure in which two Pvg1p molecules formed an asymmetric unit with two-fold axis (r.m.s. deviation for the 328 Cα atoms is 0.16 Å; Fig. 1). Approximately 928 Å^2^ of the surface area was buried between the dimer, and it was stabilized by many hydrophobic and hydrogen-bond forming interactions between three α-helices and a β-sheet (α1, α6, α11 and β8) (Figs 1 and 2). The N-termini of both protomers were on one side of the dimer (Fig. 1e). This orientation is consistent with a type II membrane-bound protein where both protomers would be anchored to the membrane via their N-terminal transmembrane domains. The PISA analysis^27^ suggested that the dimer is stable in solution. Because the predicted active sites were located far away from the dimer interface, the dimerization of Pvg1p seems to be not essential for the catalysis and substrate binding.

**Figure 3:**
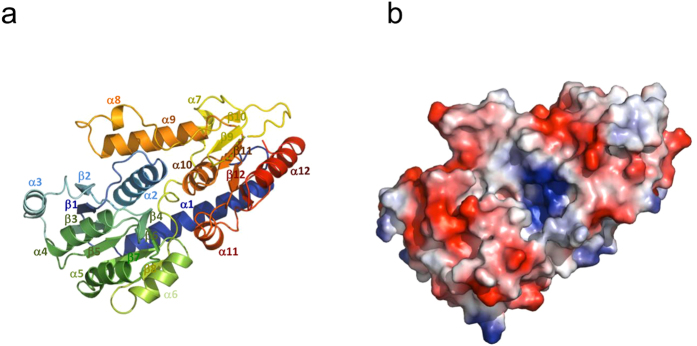
Overall structure of Pvg1p. Structure of Pvg1p is displayed using (**a**) ribbon and (**b**) electrostatic surface models. (**a**) In the ribbon model, N- and C-terminal regions are shown in blue and red, respectively. (**b**) In the electrostatic surface model, positive and negative charges are depicted in blue and red, respectively.

The crystal structure of Pvg1p consisted of twelve α-helices and twelve β-sheets, and two α/β/α domains at the N- and C-terminal half regions (Figs 2 and 3a). Using the NCBI Vector Alignment Search Tool program (VAST), we found that the structure of glycosyltransferase MshA of *Corynebacterium glutamicum*^28^ (PDB, 3C4V) resembled that of Pvg1p very well, even though there exists no sequence homology between these two proteins (Supplementary Fig. S2). MshA belongs to type-B glycosyltransferases (GT-B) and contains GT-B fold. Like Pvg1p^26^, MshA does not require any metal ion for its activity^29^.

### Proposed catalytic mechanism based on enzyme-substrate complex modeling and complementary mutational analyses of Pvg1p

**Figure 4:**
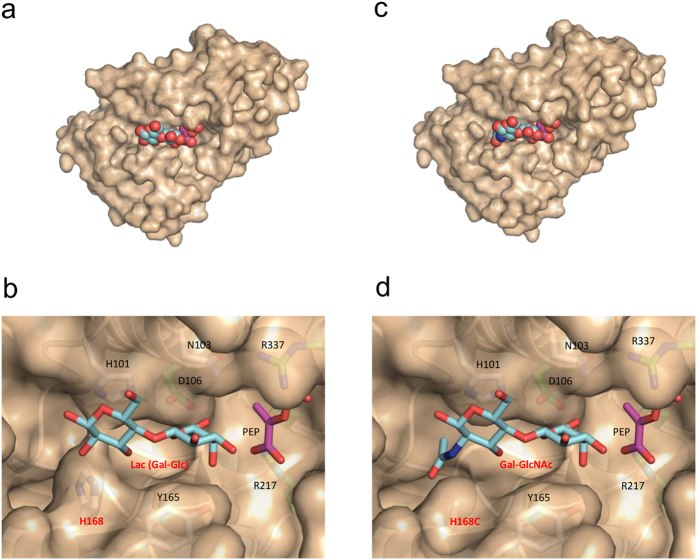
Predicted ES-complex structures of Pvg1p and Pvg1p^H168C^. (**a**) Overall structure of the predicted ES-complex Pvg1p-PEP-Lac. (**b**) Predicted active site structure of the ES-complex Pvg1p-PEP-Lac. (**c**) Overall structure of the predicted ES-complex Pvg1p^H168C^-PEP-Gal-GlcNAc. (**d**) Predicted active site structure of the ES-complex Pvg1p^H168C^-PEP-Gal-GlcNAc.

Though we tried to co-crystallize Pvg1p with its two substrates (PEP and β-Gal-*p*NP), both substrates were not present in the active site. Using the apo Pvg1p crystal structure, we built a substrate-bound model of Pvg1p using PEP as a donor substrate and lactose (Lac; Gal-β1,4-Glc) as an acceptor substrate. We used Lac for our model building because it mimics the β-Gal residue of the native oligosaccharide substrate of *S. pombe* (Fig. 4a,b). Charged surface representation analysis clearly showed a positively charged cleft situated between the N- and C-terminal halves of Pvg1p, which is suitable for the binding of negatively charged donor substrate PEP (Fig. 3b). In this model, PEP and Lac bound deep into this cleft. Remarkably, the predicted binding sites for the substrates of Pvg1p are almost same as the binding sites for the substrates of MshA (Supplementary Fig. S2), even though the substrates as well as the reactions of these two enzymes are different. This observation is also consistent with our prediction. In the proposed model, only the D106 residue is situated within hydrogen bond forming distance of the O6 oxygen of the galactose residue of Lac, whereas both R217 and R337 residues directly interact with PEP (Fig. 4b, Supplementary Fig. S3). It is interesting to note that all three residues are completely conserved in Pvg1p homologs (Fig. 2). Therefore, these three residues are potential candidates of catalytic residues for the pyruvylation reaction.

To test the reliability of this predicted model, we performed mutational analysis of Pvg1p. For this purpose, we tried to replace D106, R217 and R337 residues of Pvg1p individually with an alanine (A) residue by mutagenesis and obtain three point mutants: Pvg1p^D106A^, Pvg1p^R217A^ and Pvg1p^R337A^. Although we could express recombinant Pvg1p^D106A^, we failed to express Pvg1p^R217A^ and Pvg1p^R337A^ in *E. coli*. Consistent with the above-mentioned predicted model, the purified Pvg1p^D106A^ showed almost no relative enzyme activity using β-Lac-*p*NP as an acceptor substrate.

### Design of Pvg1p that efficiently transfers the pyruvyl group to the terminus of human-type complex oligosaccharide

Since sialic acid attachment also provides negative charge on the cell surface, the effects of pyruvylation and sialylation of oligosaccharides are likely commensurate with their functions. Therefore, we next explored the possibility of producing pyruvyl human-type complex glycopeptide using Pvg1p. First, we examined whether Pvg1p could use *p*NP-attached Gal-α1,4-GlcNAc (LacNAc), which is normally found at the end of human-type complex glycan chains, as a substrate. We found that the wild-type Pvg1p has very low pyruvyltransferase activity for the LacNAc-*p*NP (Supplementary Fig. S4a). Based on our predicted substrate-binding model of Pvg1p, less pyruvylation was probably due to the steric hindrance posed by the NAc group of the LacNAc-*p*NP with the H168 of Pvg1p (Fig. 4d), as a result of which it could not bind properly to the active site of Pvg1p. Thus, we created the H168A mutant of Pvg1p (Pvg1p^H168A^) to examine whether this Pvg1p mutant would recognize the human-type complex oligosaccharides. For this purpose, we expressed recombinant Pvg1p^H168A^ in *E. coli* and successfully purified it. Remarkably, Pvg1p^H168A^ exhibited higher activity for transferring pyruvyl group to LacNAc-*p*NP than the wild-type Pvg1p (Supplementary Fig. S4b), suggesting that Pvg1p^H168A^ may be able to add pyruvate on the human-type complex oligosaccharides.

Although Pvg1p^H168A^ could add pyruvate to LacNAc-*p*NP, the activity was not ideal to obtain sufficient amount of pyruvylated oligosaccharide for further experiments. Therefore, to obtain a Pvg1p mutant with better pyruvyltransferase activity, we performed extensive mutational analysis of the H168 residue of Pvg1p by individually replacing it with the remaining 18 amino acid residues. Among all mutants, the H168C mutant of Pvg1p (Pvg1p^H168C^) exhibited highest activity for transferring pyruvate onto LacNAc-*p*NP (Supplementary Table S1). Consistent with these results, we confirmed by model building that LacNAc-*p*NP fitted well in the active site of the predicted model of Pvg1p^H168C^ (Fig. 4c,d).

### Generation and assessment of neo pyruvyl human-type complex glycopeptide

**Figure 5:**
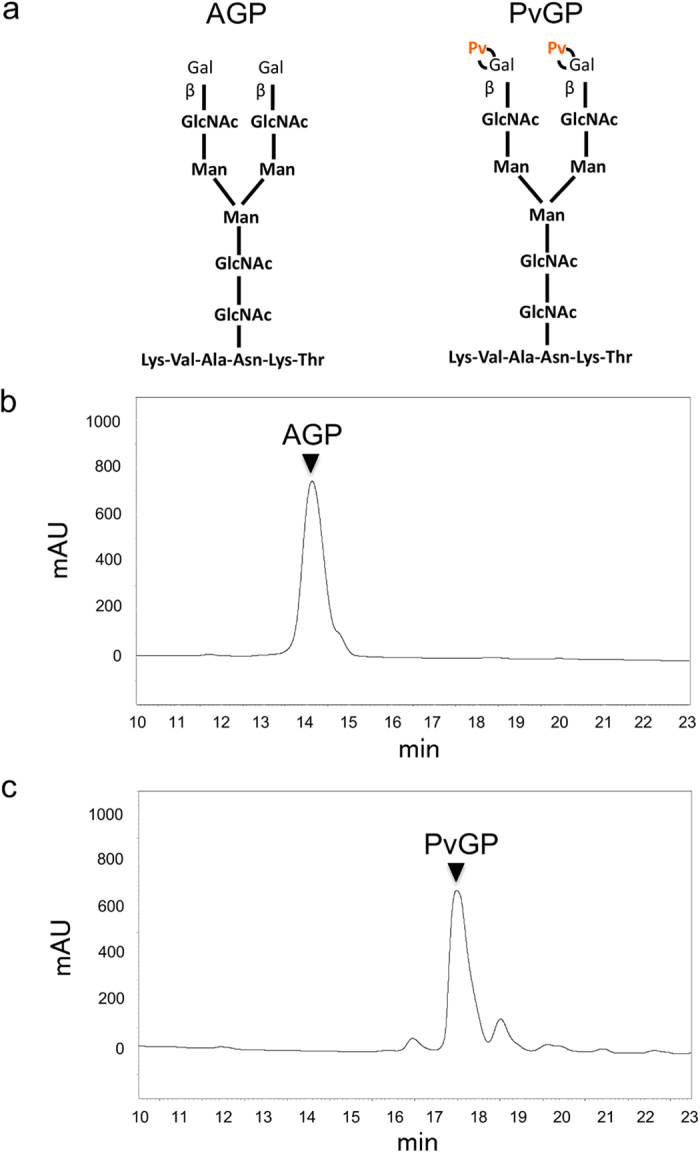
Generation of pyruvyl neo-human-type complex glycopeptide. (**a**) Structure of the glycopeptides AGP (left, no pyruvylation) and PvGP (right, both ends of the oligosaccharide chains are pyruvylated). HPLC chromatograms of (**b**) AGP and (**c**) PvGP. AGP was treated without (**b**) or with (**c**) Pvg1p^H168C^ and reaction mixtures were analyzed by HPLC. Sample collected from the highest peak in (**c**) indeed contained PvGP, which was confirmed by mass spectrometry analysis (see Supplementary Fig. S5).

We next determined whether Pvg1p^H168C^ could attach pyruvate onto both terminal β-Gal residues of asialo glycopeptide (AGP), a human-type complex oligosaccharide acceptor substrate (Fig. 5a). HPLC analysis of the reaction mixture revealed that the retention time of the resulting reaction product was different from that of AGP (compare Fig. 5b,c), suggesting that the pyruvate moiety became attached to the terminal oligosaccharide chain of AGP. To confirm that biantennary pyruvyl glycopeptide (PvGP) was indeed produced by this reaction, the peak sample obtained from the HPLC analysis was collected and analyzed by MALDI-TOF MS. As can be seen from the mass spectrum, the observed m/z value was in good agreement with the calculated molecular mass of PvGP (observed, m/z = 2421.053; calculated, [M−N] = 2421.36) (Supplementary Fig. S5), which confirmed that the peak sample collected from HPLC analysis mainly consisted of PvGP.

### Pyruvylation bestows lectin binding properties similar to α2,6-sialylation

**Figure 6:**
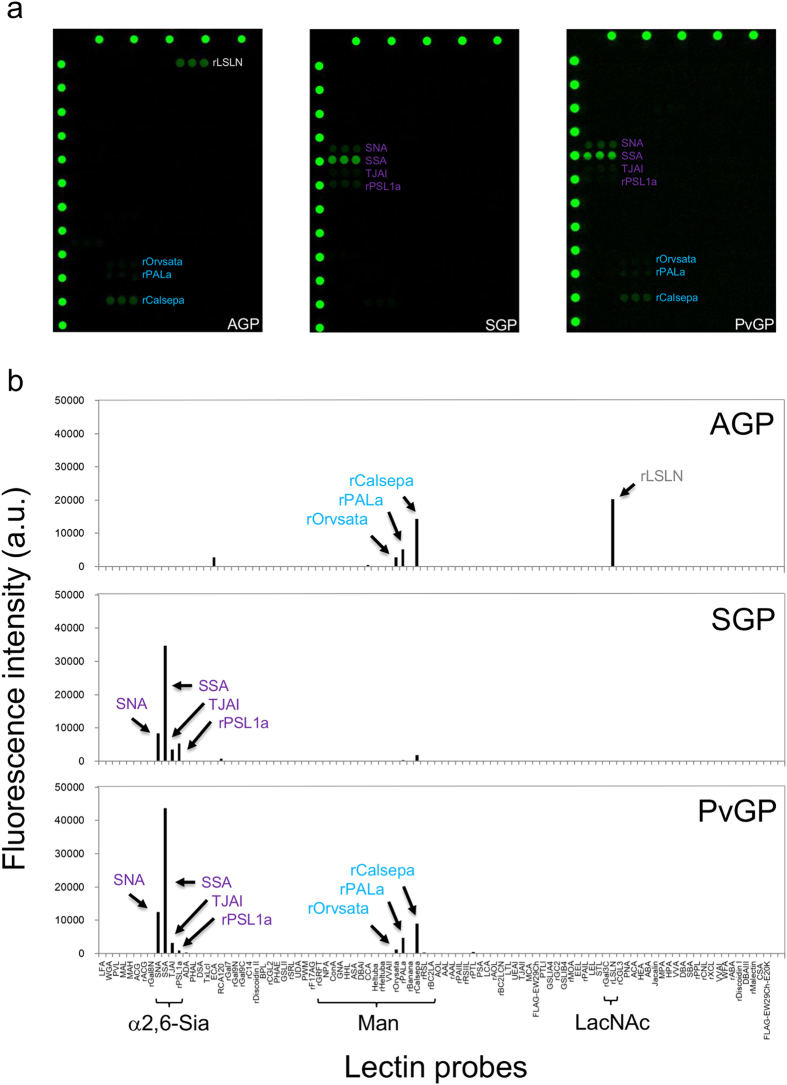
Lectin array analysis of asialo, sialyl and pyruvyl human-type complex glycopeptides. (**a**) Fluorescence detection images of lectin arrays using asialo (AGP), sialyl (SGP) and pyruvyl (PvGP) human-type complex glycopeptides. (**b**) Quantified fluorescence intensities in lectin array using AGP, SGP or PvGP. Note that the array results obtained using PvGP exhibited characteristics of array results of both AGP and SGP.

Next, we tested whether pyruvylated human-type complex oligosaccharides would exhibit characteristics similar to the sialylated ones. To achieve this, we performed a lectin array analysis^30^, which can detect interactions between a target oligosaccharide and varieties of lectins (Fig. 6a). For a positive control, we first confirmed that sialyl glycopeptide (SGP), in which two sialic acid molecules are attached to both ends of oligosaccharide chains, displayed affinity for binding to α2,6-sialic acid-binding lectins (such as SNA, SSA, TJAI and rPSL1a), but not to α2,3-sialic acid-binding lectins (such as MAL, MAH, ACG, rACG and rGal8N); glycopeptide AGP was used as a negative control for this experiment (Fig. 6a). Interestingly, lectin binding properties of PvGP were very similar to those of SGP (i.e., PvGP bound to α2,6-sialic acid-binding lectins, but not to α2,3-sialic acid-binding lectins) (Fig. 6a). In addition, as shown in Fig. 6b, PvGP displayed affinity for binding to mannose lectins (rOrvsata, rPALa and rCalsepa); AGP, but not SGP, also exhibited similar affinity for binding to mannose lectins. Since sialic acid moiety of SGP is larger than the pyruvate moiety of PvGP, it is possible that the mannose residues are masked in SGP, but not in PvGP and AGP. Furthermore, PvGP, like SGP, did not exhibit any affinity for binding to a LacNAc lectin rLSLN, suggesting that the β-Gal residues were probably masked by the pyruvate moiety in PvGP. Collectively, these results suggest that as far as the lectin binding is concerned, both pyruvylation and α2,6-sialylation confer similar characteristics to human-type complex oligosaccharides.

Finally, we performed experiments to analyze whether PvGP would be resistant to sialidase digestion. As a control, SGP was treated with a sialidase derived from *Arthrobacter ureafaciens*, which has α2,6-sialidase activity. Thus, treatment of SGP with sialidase resulted in AGP (Supplementary Fig. S6a,b). In contrast, sialidase treatment did not cause any change in PvGP, as the retention time following the sialidase treatment remained same (Supplementary Fig. S6c,d), suggesting that PvGP is resistant to α2,6-sialidase digestion.

## Discussion

The fission yeast pyruvyltransferase Pvg1p, which is localized to the Golgi membrane, attaches pyruvate moieties on oligosaccharides and consequently introduces negative charges on the cell surface. These cell surface negative charges play significant physiological roles, such as intercellular communication. In this study, we determined the crystal structure of Pvg1p at a resolution of 2.46 Å, and based on the crystal structure, we rationally designed a Pvg1p that exhibited altered substrate preference. Thus, we created a mutated Pvg1p, Pvg1p^H168C^, that can efficiently add pyruvate onto a human-type complex glycopeptide, and this pyruvylated glycopeptide showed lectin binding properties similar to those of the α2,6-sialylated glycopeptide.

Intriguingly, the crystal structure of Pvg1p resembles the structures of GT-B fold harboring glycosyltransferases, including sialyltransferase^31^ (Supplementary Fig. S7). Although no amino acid sequence homology has been found between Pvg1p and any GT-B type glycosyltransferase, it is possible that these enzymes may have evolved from a common enzyme. In any event, the observed structural similarities between the transfer enzymes and characteristic similarities between the modified oligosaccharides suggest an evolutionary relationship between pyruvylation and sialylation.

Pyruvylation onto human-type complex oligosaccharides might find some useful applications. For example, to heterologously produce human proteins in yeast, glycan modification is a crucial factor to consider^32^, as lack of proper glycan modification could lead to the synthesis of an inactive protein. *Pichia pastoris*, which is widely used for the heterologous expression of human proteins, lacks this glycan modification system. Thus, to generate a *P. pastoris* strain that could produce bioactive human-type complex glycoproteins, it would require introduction of several genetic components, including the genes for sialyltransferase as well as the genes for substrate supply^33^. In contrast, if indeed pyruvylation could mimic sialylation, as the results of this study seem to indicate, then we would only need a Pvg1p^H168C^ expressing *S. pombe* strain, because all other components, including the genes for substrate synthesis as well as substrate supply are already present in these cells. Another possible application of pyruvylation is that since hemagglutinin (HA) of influenza virus recognizes sialic acid on oligosaccharides of cell surface, pyruvylation might be able to competitively inhibit the binding between HA and sialic acid, resulting in the prevention of influenza virus infection. This principle could serve as a useful strategy for providing a new class of anti-influenza virus drugs.

Pyruvylated neo-human-type complex glycoproteins might also mimic sialyl glycoproteins *in vivo*. Since desialylation by sialidase is a signal for initiating the degradation process of glycoproteins in hepatic cells^34^,^35^, it might be possible that pyruvylation would help in circumventing the degradation problem of glycoproteins that are used as drugs. In fact, we have demonstrated in this study that the pyruvylated glycopeptide PvGP not only exhibits characteristics similar to those of SGP, as assessed by lectin microarray analysis, but unlike SGP it also shows resistance to sialidase digestion. Further *in vivo* kinetic analysis would be required to determine whether pyruvylated pharmaceutical glycoproteins would have improved half-life. Recently, several methodologies have been developed to produce pharmaceutically useful glycoproteins with longer half-life. For example, the production of erythropoietin (EPO) was improved by increasing its sialic acid content^36^. Another recent study reported that modification of sialic acid of EPO conferred resistance to sialidase^37^, suggesting that modification by pyruvylation might also confer similar resistance to sialidase. Thus, detailed characterization of the pyruvylated oligosaccharides would further broaden our understanding about the effects of pyruvylation and would also facilitate the progress in glycobiology research fields, especially for developing glycoprotein based pharmaceutical agents.

## Methods

### Plasmids and strains

For all recombinant DNA procedures, *E. coli* strain XL-1 Blue (Stratagene) was used. To express each point mutant (generated by a PCR-based mutagenesis method^26^), an expression plasmid harboring the point mutant was created by replacing the open-reading frame (ORF) of the wild-type Pvg1p in plasmid pET32b-*pvg1* with the ORF of the Pvg1p point mutant. Primers used to generate the point mutants of Pvg1p are summarized in Table S2. Recombinant proteins (wild-type and mutant Pvg1ps) were purified as described before^25^ and used in enzyme assay.

### Crystallization

Recombinant Pvg1p was purified as described previously^25^ with the following modifications. Ni-affinity and gel filtration chromatographic procedures were performed using a HisTrapTM FF (1 mL) column and a Superose 6 10/300 GL column (GE Healthcare), respectively. Single crystals were grown by the hanging-drop vapor diffusion method at 20 °C. The protein solution was composed of 10 mg/mL Pvg1p in 100 mM MOPS-NaOH (pH 7.4) containing 20 mM PEP and 5 mM *p*NP-β-Gal. The well solution contained 200 mM magnesium choloride, 10 mM zinc chloride, 26% (w/v) Poly(acrylic acid sodium salt) 5100 in 0.1 M HEPES (pH 7.5) buffer. Each hanging-drop consisted of 1 μL of protein solution and 1 μL of well solution.

### Data collection

A single crystal was transferred from the mother liquor to a cryoprotectant solution consisting of 20 mM magnesium chloride, 0.1 M HEPES, pH7.5, 26% Poly(acrylic acid sodium salt) 5100, 10 mM zinc chloride, 20 mM PEP, 5 mM *p*NP-β-Gal, 0.1 M MOPS-NaOH, pH7.4, and 25% glycerol. The crystal was mounted on a cryo-loop, and flash-cooled with a stream of nitrogen gas 100 K using a cryosystem (Rigaku). X-ray diffraction data were collected using ADSC Q210 and synchrotron radiation (1.2817 Å wavelength) at the beamline BL38B1 of SPring-8 (Hyogo, Japan). Diffraction data were processed using the program package HKL2000^38^. Data collection statistics are summarized in Table 1. A phenix xtriage software indicated that the data was partial twin^39^. The estimated twin law was (h, -h-k, -l) and estimated twin fraction was 0.24.

### Structure determination and refinement

The initial phase structure of the Zn^2+^ binding crystal of Pvg1p was determined by the single-wavelength anomalous diffraction (SAD)^40^ method and using the Phenix software package^41^,^42^ with twin data option. Twin law (h, -h-k, -l) was used for phase determination and refinement process. The partial mode was built automatically using the Phenix software and modified manually using COOT^43^. The structure was revised several times by alternately adjusting the model and refinements were made using Refmac^44^. Though Pvg1p dimer existed in the asymmetric unit, the noncrystallographic axis was not parallel to the twinning axis. Typical electrondensity map was shown in Supplementary Fig. S8. The Zn^2+^ bound between dimer-dimer interface in the crystal packing (Supplementary Fig. S9). The refinement statistics are summarized in Table 1. Stereochemical checks were carried out using Molprobity^45^. The atomic coordinates and structural parameters of the wild-type and iodine-substituted protein have been deposited in the Protein Data Bank at Rutgers University under accession code 5AX7. All crystal structure related figures were prepared using PyMol (http://pymol.sourceforge.net).

### Modeling of Pvg1p-PEP-Lac complex structure

A molecular model of Pvg1p-PEP-Lac complex was constructed using MOE (2014.09; Chemical Computing Group Inc., Montreal, Canada). First, PEP was placed into the predicted substrate-binding pocket of Pvg1p using ASEDock option of MOE. After energy minimizaion of the PEP-bound complex, Lac was placed into the substrate-binding pocket of the Pvg1p-PEP complex structure by following the same procedure. The structure of PEP and Lac bound Pvg1p was then energy minimized. All protein atoms were fixed during the energy minimization process. The same procedure was used to construct the models for the Pvg1p^H168C^-PEP-Gal-GlcNac complex. To analyze nonbonded interactions between Pvg1p and the modeled ligands PEP and Lac, ligplot program was utilized^46^.

### Analysis of Pvg1p activity

The relative activity of Pvg1p (wild-type and mutant) was analyzed by HPLC and the assay was performed essentially as reported earlier^25^, using PEP as a donor substrate and β-Lac-*p*NP or AGP as an acceptor substrate.

### Preparation of neo-human-type complex glycopeptide modified with pyruvate

To generate a pyruvate-containing human-type glycopeptide, AGP (H-Lys-Val-Ala-Asn[(Gal-GlcNAc-Man)_2_-Man-GlcNAc_2_]-Lys-Thr-OH; Fushimi Pharmaceutical) was used as an acceptor substrate. In 150 μL of 0.2 M MOPS-NaOH buffer (pH 7.5), 0.5 mg recombinant Pvg1p^H168C^ was incubated with 40 mM PEP monopotassium salt and 1 mg AGP at 30 °C for 16 h. After stopping the reaction by boiling and removing particulate matters by centrifugation, the supernatant was subjected to HPLC analysis using a GL-7400 HPLC system equipped with a UV spectrophotometer (GL Sciences) and a Wakosil 5C18 reverse-phase column (Wako, 4.6 × 250 mm) set at 30 °C. For separation, 0.1% trifluoroacetic acid (TFA) and 100% acetonitrile were used as mobile phases at a flow rate of 1 mL/min and the eluate was monitored at 215 nm. Sialidase (Sanyo Fine Co., Ltd.) treatment of SGP (Fushimi Pharmaceutical) and PvGP were carried out according to the manufacturer’s instructions, and the resultant products were analyzed by HPLC as described above.

### MALDI-TOF MS analysis

MALDI-TOF MS analysis was carried out using Autoflex II (Bruker Daltnics). The HPLC-purified sample was dissolved in water at a concentration of 1 μg/μL, which was then placed on an anchoring plate and covered with matrix solution (5 mg/mL 2,5-dihydroxybenzoic acid in a 2:1 mixture of 0.1% TFA:acetonitrile). After drying the plate, it was used for the MS analysis in the negative ion mode.

### Lectin microarray analysis

Lectin microarray analysis was performed essentially as described previously^30^. AGP and SGP were used as controls. Each glycopeptide sample was labeled with Cy3 dye and concentration of the sample was set at 1 μg/mL. It was then applied to the lectin microarray plate (60 μL/well) and incubated overnight at 20 °C. After washing the plate with the probing buffer (140 mM NaCl, 2.7 mM KCl, 1 mM CaCl_2_, 1 mM MnCl_2_, 1% Triton X-100 and 25 mM Tris-HCl, pH 7.5), fluorescent images were captured using an evanescent field-activated fluorescent scanner (GP BioSciences). The fluorescent intensity of each well was measured using Array Pro Analyzer version 4.5 (Media Cybernetics, Bethesda, MD) after subtraction of the background value, which was obtained from the fluorescent intensity of the well containing no lectin sample. For quantitative analysis, signal intensities obtained from triplicate wells were averaged.

## Additional Information

**How to cite this article**: Higuchi, Y. *et al*. A rationally engineered yeast pyruvyltransferase Pvg1p introduces sialylation-like properties in neo-human-type complex oligosaccharide. *Sci. Rep*. **6**, 26349; doi: 10.1038/srep26349 (2016).

## Supplementary Material

Supplementary Information

## Figures and Tables

**Table 1:** Data collection and refinement statistics.

**Data collection**	
Space group	*P*3_1_
Unit cell parameters (Å)	a = b = 85.6, c = 93.6
Wavelength (Å)	1.2817
Resolution range (Å)	10.99–2.46 (2.52–2.46)
No. of reflections	
Observed/Unique	321427/55667
Redundancy	5.77 (5.51)
*R*_sym_^a,b^	0.062 (0.654)
*I*/σ (*I*)^a^	25.14 (2.52)
Completeness (%)	99.8.0 (98.8)
Estimated Twin law	h, -h-k, -l
Estimated Twin fraction (α)	0.24
**Refinement statistics**	
Resolution range (Å)	10.99–2.46
No. of reflections	
Working set/Test set	26299/1461
Completeness (%)	98.84
*R*_cryst_^c^ (%)/*R*_free_^d^ (%)	16.73/18.34
Root mean square deviation	
Bond length (Å)	0.005
Bond angles (°)	0.959
Average B-factor (Å^2^)/No. of atoms	
Protein	54.2/5257
Water	45.4/67
Ramachandran analysis	
Favored (%)	95.9
Allowed (%)	3.2
Outlier (%)	0.9
