# Supplementary material for: A rationally engineered yeast pyruvyltransferase Pvg1p introduces sialylation-like properties in neo-human-type complex oligosaccharide
Source: Sci Rep. 2016 May 19;6:26349. doi: 10.1038/srep26349 (PMC4872226; doi:10.1038/srep26349)

Supplementary Information

A rationally engineered yeast pyruvyltransferase Pvg1p introduces sialylation-like properties in neo-human-type complex oligosaccharide

Yujiro Higuchi[1], Sho Yoshinaga[1], Ken-ichi Yoritsune[1], Hiroaki Tateno[2], Jun Hirabayashi[2], Shin-ichi Nakakita[3], Miho Kanekiyo[1], Yoshimitsu Kakuta[1,*], Kaoru Takegawa[1,*]

[1]Department of Bioscience and Biotechnology, Faculty of Agriculture, Kyushu University, 6-10-1 Hakozaki, Fukuoka 812-8581, Japan

[2]Biotechnology Research Institute for Drug Discovery, National Institute of Advanced Industrial Science and Technology, Central-2, 1-1-1, Umezono, Tsukuba, Ibaraki 305-8568, Japan

[3]Department of Functional Glycomics, Life Science Research Center, Kagawa University, Miki-cho, Kagawa 761-0793, Japan

[*]Corresponding authors. Tel/Fax: +81 92 642 7159, E-mail address: kakuta@agr.kyushu-u.ac.jp (Y. Kakuta); Tel/Fax: +81 92 642 2851, E-mail address: takegawa@agr.kyushu-u.ac.jp (K. Takegawa)

Number of supplementary figure, 9.

Number of supplementary table, 2.

Supplementary Figure Legends

Figure S1. Purification of recombinant Pvg1p.

Recombinant Pvg1p was expressed in *E. coli* and purified first by Ni-affinity chromatography followed by gel filtration chromatography. The resultant protein was analyzed by SDS-PAGE and the gel was subsequently stained with CBB. Mr, protein molecular weight markers.

Figure S2. Comparison of crystal structures of Pvg1p and MshA.

The crystal structure of Pvg1p (a) shows highest structural similarity to that of *Corynebacterium glutamicum* MshA (b). (c) Superposition of the structure of Pvg1p (orange) with that of MshA (gray) complexed with UDP and *L*-Myo-inositol-phosphate.

Figure S3. Ligplot diagram of the nonbonded interactions.

Ligplot diagrams of the nonbonded interactions between the modeled ligands (PEP and Lac) and Pvg1p in the active site are depicted.

Figure S4. Pyruvyltransferase activity of Pvg1p$^{\mathrm{H168A}}$.

Pyruvyltransferase assay was performed using either (a) recombinant wild-type or (b) recombinant H168A mutant of Pvg1p (Pvg1p$^{\mathrm{H168A}}$) as the enzyme and LacNAc-*p*NP as an acceptor substrate. Each reaction mixture was analyzed by HPLC. Note that a new peek (labeled with arrowhead 1) was observed in the HPLC chromatogram of the reaction mixture that contained Pvg1p$^{\mathrm{H168A}}$ (b). Arrowhead 2 indicates the peak of LacNAc-*p*NP that appeared on the HPLC chromatogram.

Figure S5. Confirmation of the identity of HPLC-separated sample by MALDI-TOF

MS analysis.

MALDI-TOF MS analysis of the sample corresponding to the peak on the HPLC chromatogram (Figure 5c). The observed m/z value is consistent with the calculated molecular mass of biantennary pyruvyl glycopeptide, PvGP.

Figure S6. HPLC analyses of SGP and PvGP after sialidase treatment.

(a) SGP without sialidase treatment. (b) SGP treated with sialidase, producing AGP. (c) PvGP without sialidase treatment. (d) PvGP treated with sialidase. Note that unlike SGP, PvGP was not hydrolyzed by sialidase.

Figure S7. Comparison of crystal structures of Pvg1p and α2,6-sialyltransferase.

(a) Crystal structure of Pvg1p. (b) Pvg1p-like structural areas of *Photobacterium* sp. α2,6-sialyltransferase complexed with bound CMP and lactose (PDB, 2Z4T). (c) Superposition of the structure of Pvg1p (orange) with the structurally similar areas of the α2,6-sialyltransferase (blue) complexed with CMP and lactose.

Figure S8. Electron density maps of Pvg1p.

(a) Final 2Fo-Fc electron density map contoured at 1 σ of the β-sheet area of Pvg1p. (b) Stereo 2Fo-Fc electron density maps of the active site (2.0 σ).

Figure S9. Zn^{2+} binding sites of Pvg1p.

(a) Crystal packing of Pvg1p with Zn^{2+}. (b) Zn^{2+} binding site between the dimer-dimer interface. 2Fo-Fc electron density map (2.0 σ) in blue and anomalous difference map (7.0 σ) in green.

Supplementary Table S1. Relative activities of recombinant Pvg1ps.

Pvg1p	Relative activity
Wild-type (H168)	100
A	778
C	3160
D	315
E	1830
F	19.1
G	109
I	133
K	28.4
L	65.1
M	108
N	719
P	1320
Q	1980
R	80.9
S	479
T	916
V	443
W	6.10
Y	15.3

H168 residue of Pvg1p was individually mutagenized into 19 remaining amino acid residues. LacNAc-*p*NP was used as an acceptor substrate.

Supplementary Table S2. Primers used for generating point mutants of Pvg1p.

Target	Sequence (5’ to 3’; upper, forward; lower, reverse)
pvg1-D106A	5’-AAGGGTGCTAGTGCAATCTATGTTGCTGAG-3’
	5’-TGCACTAGCACCCTTGTTGGGGTGGTCAGG-3’
pvg1-R217A	5’-GTCACTGCTGATAGGCAAAGCTATGGTTTT-3’
	5’-CCTATCAGCAGTGACCAAAGTGATATTAGG-3”
pvg1-R337A	5’-ACTGATGCTCTCCATGCTCACATCCTTAGC-3’
	5’-ATGGAGAGCATCAGTAATGACGACGCGAGC-3’
pvg1-H168A	5’-CCTGACGCTCAGCATTTACGTGAACTTGTC-3’
	5’-ATGCTGAGCGTCAGGATATAAATCGCCAAA-3’
pvg1-H168C	5’-CCTGACTGCCAGCATTTACGTGAACTTGTC-3’
	5’-ATGCTGGCAGTCAGGATATAAATCGCCAAA-3’
pvg1-H168D	5’-CCTGACGATCAGCATTTACGTGAACTTGTC-3’
	5’-ATGCTGATCGTCAGGATATAAATCGCCAAA-3’
pvg1-H168E	5’-CCTGACGAACAGCATTTACGTGAACTTGTC-3’
	5’-ATGCTGTTCGTCAGGATATAAATCGCCAAA-3’
pvg1-H168F	5’-CCTGACTTTCAGCATTTACGTGAACTTGTC-3’
	5’-ATGCTGAAAGTCAGGATATAAATCGCCAAA-3’
pvg1-H168G	5’-CCTGACGGACAGCATTTACGTGAACTTGTC-3’
	5’-ATGCTGTCCGTCAGGATATAAATCGCCAAA-3’
pvg1-H168I	5’-CCTGACATTCAGCATTTACGTGAACTTGTC-3’
	5’-ATGCTGAATGTCAGGATATAAATCGCCAAA-3’
pvg1-H168K	5’-CCTGACAAACAGCATTTACGTGAACTTGTC-3’
	5’-ATGCTGTTTGTCAGGATATAAATCGCCAAA-3’

pvg1-H168L	5’-CCTGACCTGCAGCATTTACGTGAACTTGTC-3’
	5’-ATGCTGCAGGTCAGGATATAAATCGCCAAA-3’
pvg1-H168M	5’-CCTGACATGCAGCATTTACGTGAACTTGTC-3’
	5’-ATGCTGCATGTCAGGATATAAATCGCCAAA-3’
pvg1-H168N	5’-CCTGACAACCAGCATTTACGTGAACTTGTC-3’
	5’-ATGCTGGTTGTCAGGATATAAATCGCCAAA-3’
pvg1-H168P	5’-CCTGACCCGCAGCATTTACGTGAACTTGTC-3’
	5’-ATGCTGCGGGTCAGGATATAAATCGCCAAA-3’
pvg1-H168Q	5’-CCTGACCAGCAGCATTTACGTGAACTTGTC-3’
	5’-ATGCTGCTGGTCAGGATATAAATCGCCAAA-3’
pvg1-H168R	5’-CCTGACCGTCAGCATTTACGTGAACTTGTC-3’
	5’-ATGCTGACGGTCAGGATATAAATCGCCAAA-3’
pvg1-H168S	5’-CCTGACAGCCAGCATTTACGTGAACTTGTC-3’
	5’-ATGCTGGCTGTCAGGATATAAATCGCCAAA-3’
pvg1-H168T	5’-CCTGACACCCAGCATTTACGTGAACTTGTC-3’
	5’-ATGCTGGGTGTCAGGATATAAATCGCCAAA-3’
pvg1-H168V	5’-CCTGACGTGCAGCATTTACGTGAACTTGTC-3’
	5’-ATGCTGCACGTCAGGATATAAATCGCCAAA-3’
pvg1-H168W	5’-CCTGACTGGCAGCATTTACGTGAACTTGTC-3’
	5’-ATGCTGCCAGTCAGGATATAAATCGCCAAA-3’
pvg1-H168Y	5’-CCTGACTATCAGCATTTACGTGAACTTGTC-3’
	5’-ATGCTGATAGTCAGGATATAAATCGCCAAA-3’

78

Supplementary Figure S1 (Higuchi et al)

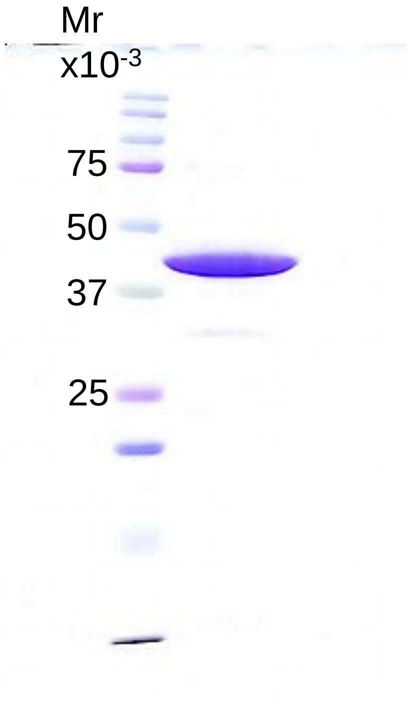

Supplementary Figure S2 (Higuchi et al)

a

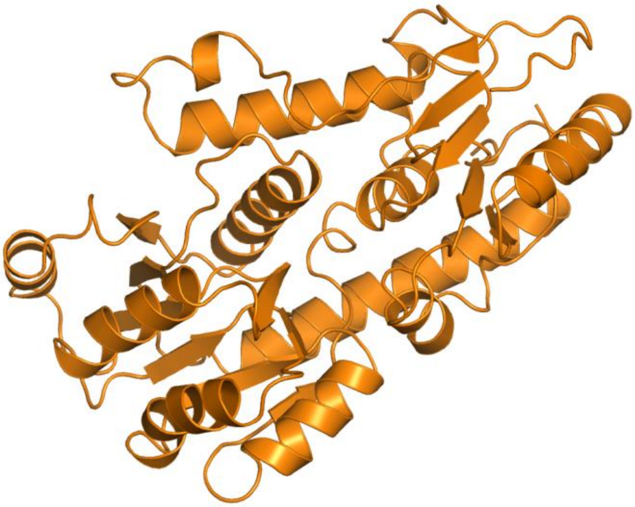

b

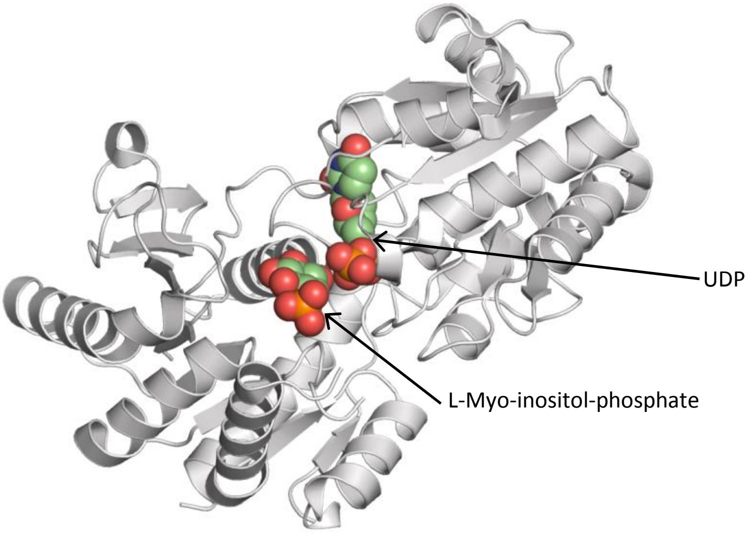

c

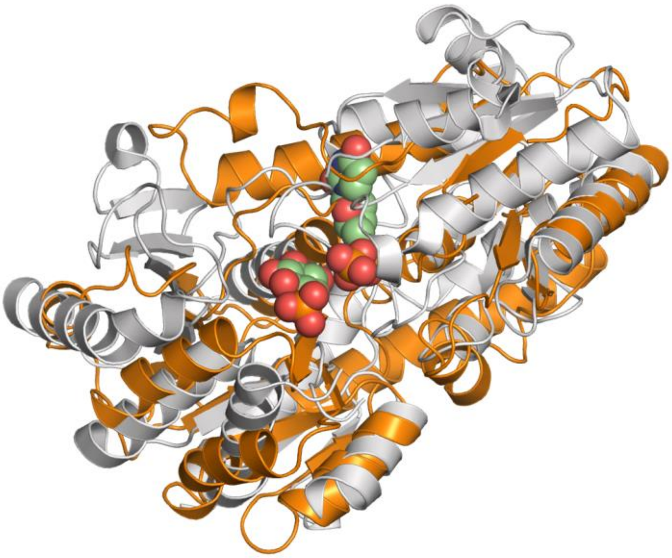

Supplementary Figure S3 (Higuchi et al)

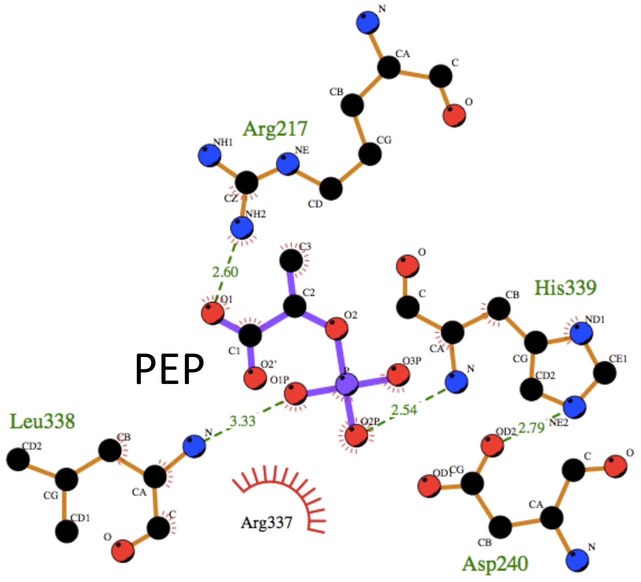

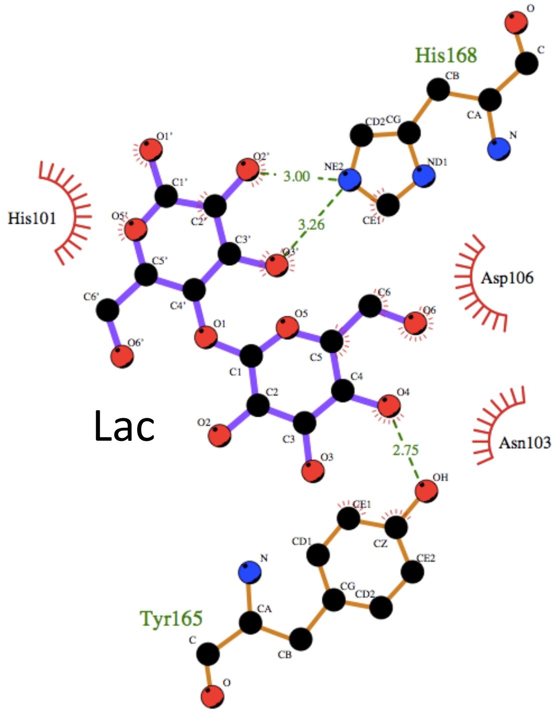

Supplementary Figure S4 (Higuchi et al)

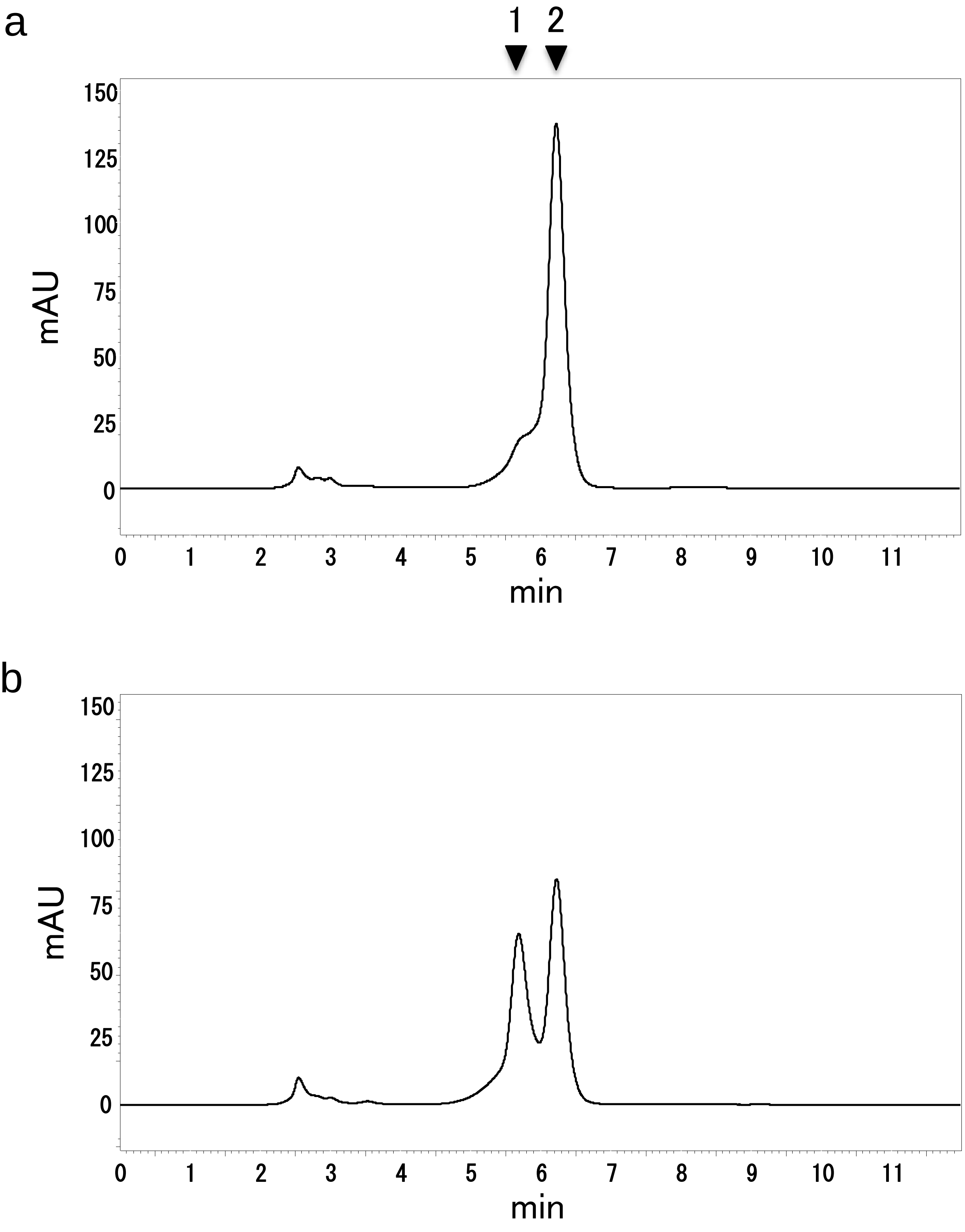

Supplementary Figure S5 (Higuchi et al)

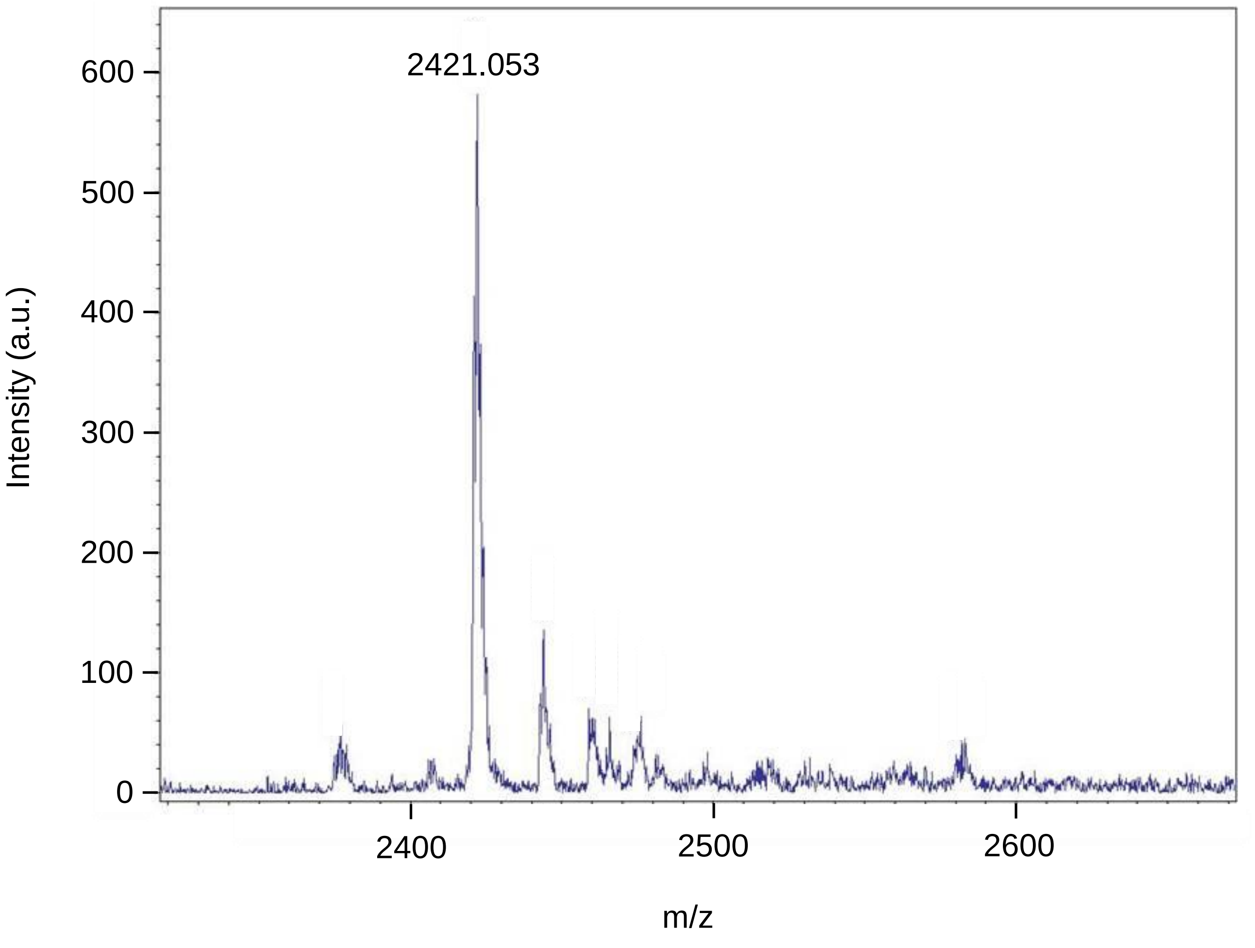

Supplementary Figure S6 (Higuchi et al)

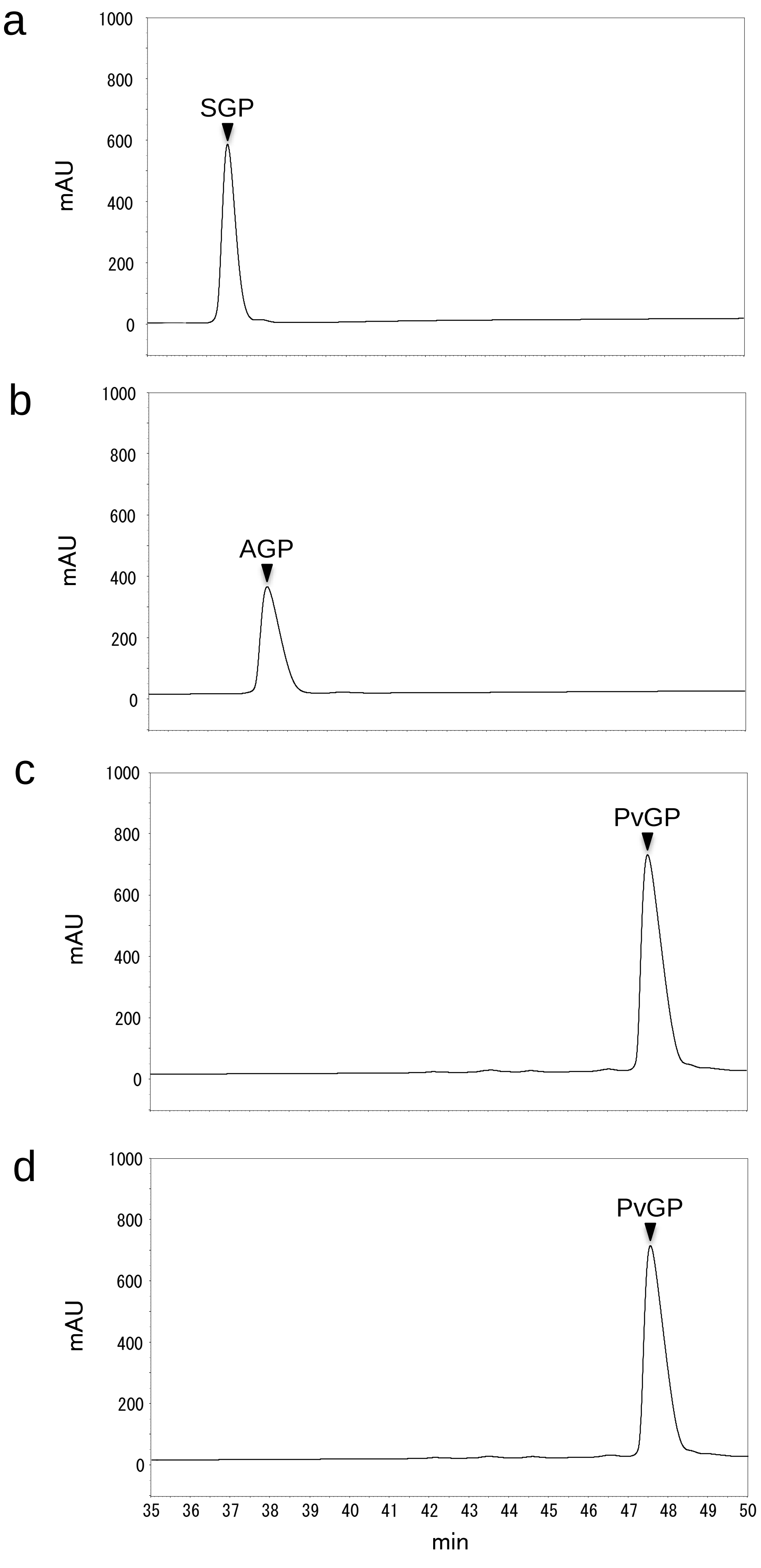

Supplementary Figure S7 (Higuchi et al)

a

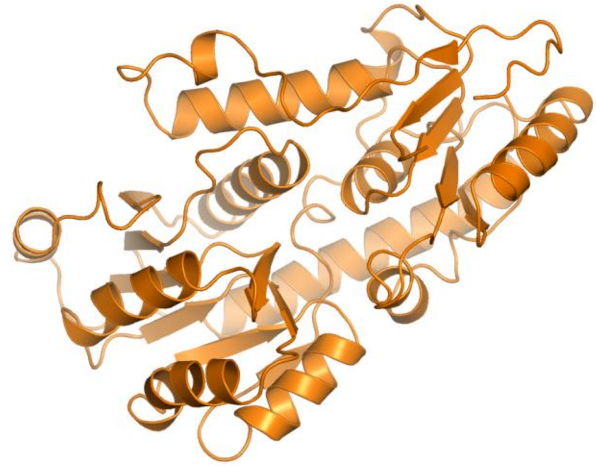

b

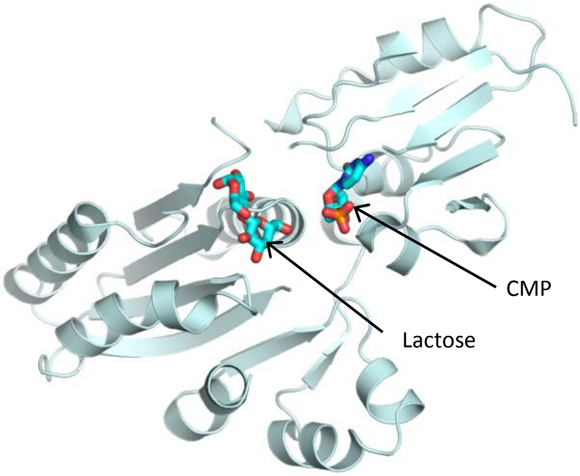

c

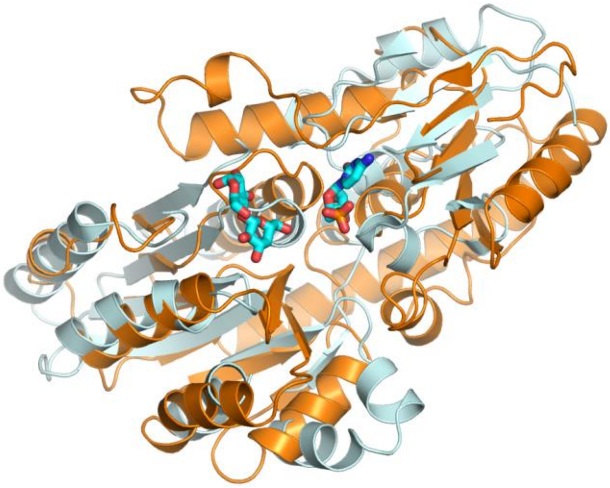

Supplementary Figure S8 (Higuchi et al)

a

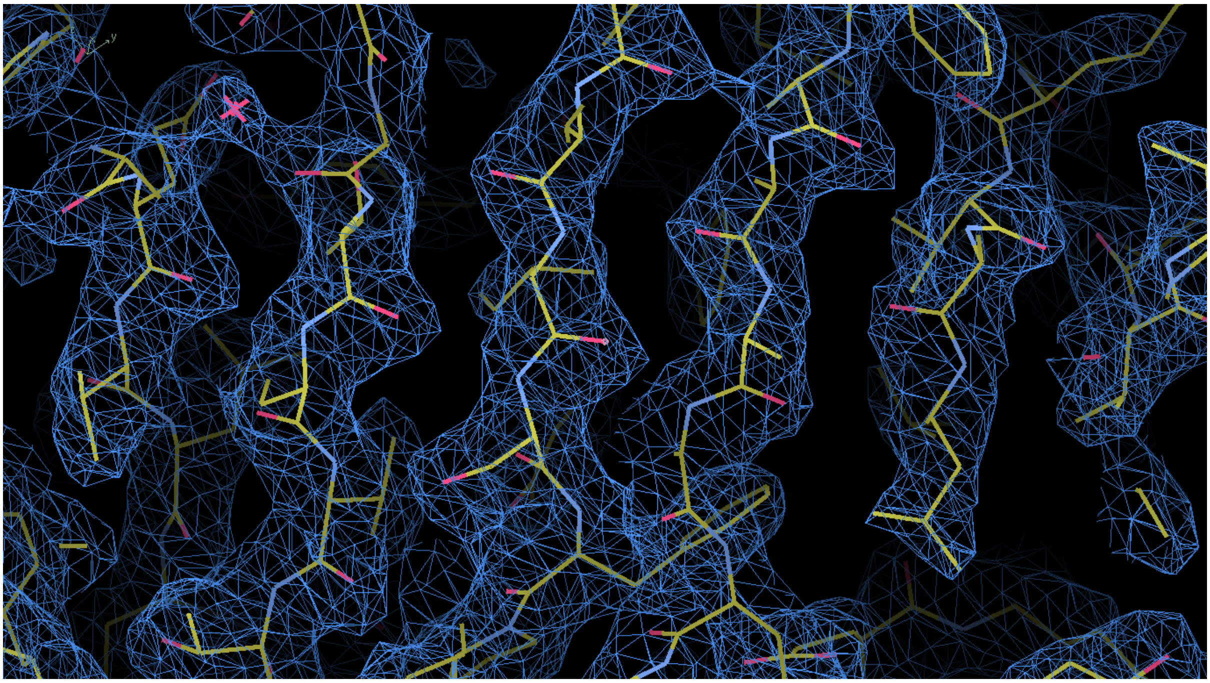

b

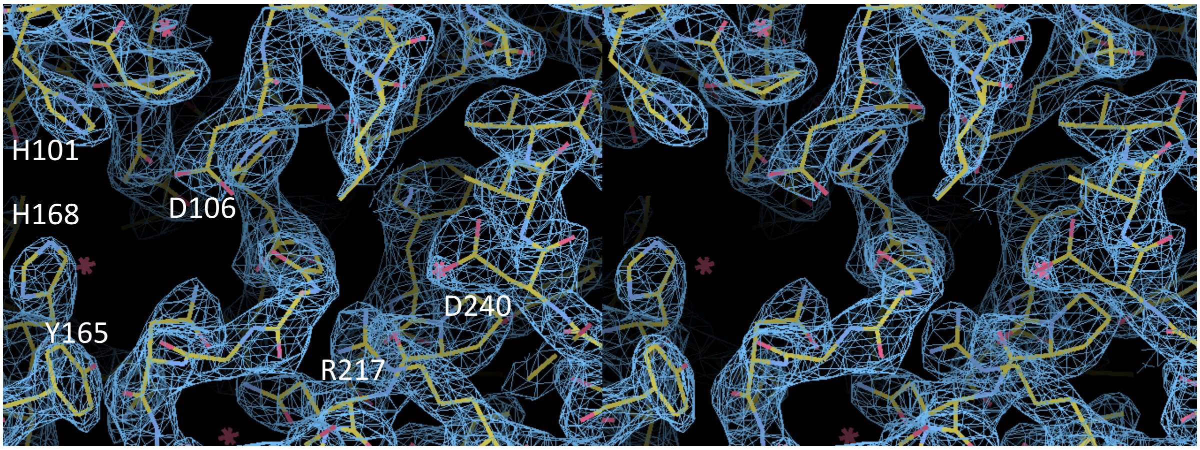

Supplementary Figure S9 (Higuchi et al)

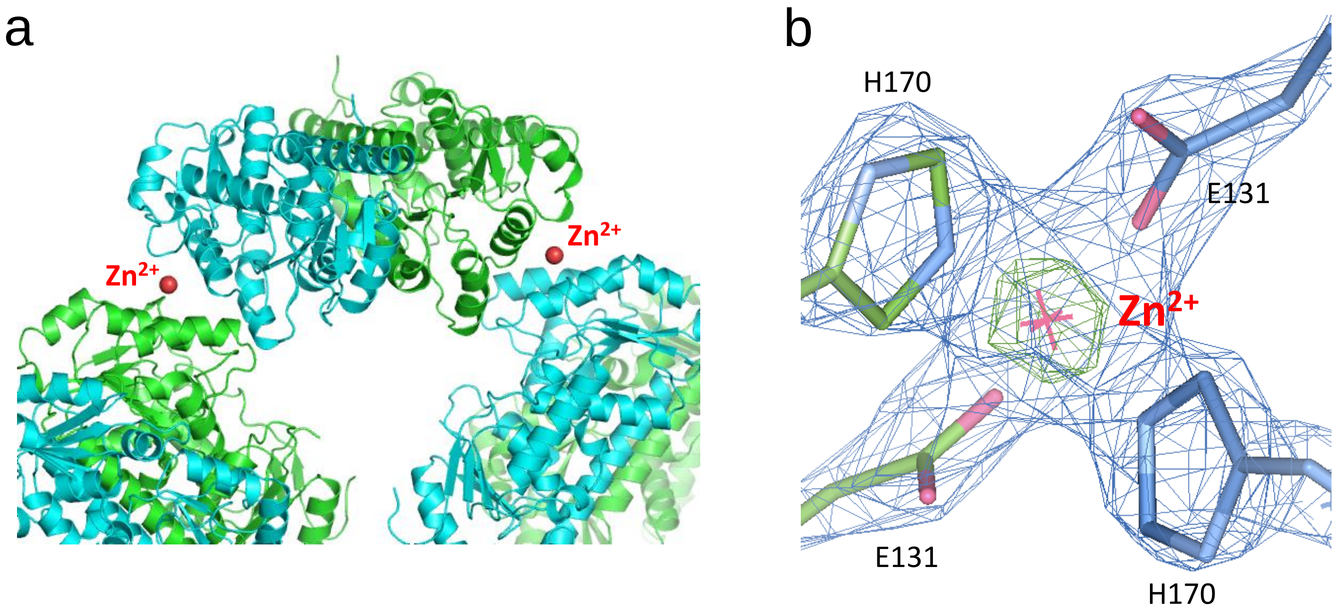

Supplement: Supplementary Information [file srep26349-s1.pdf]
